# Diagnostic value of systemic immune-inflammation index and prognostic nutritional index combined with CEA in gastric cancer with lymph node metastasis

**DOI:** 10.3389/fendo.2025.1522349

**Published:** 2025-04-14

**Authors:** Xiao-rong Dai, Min-zhe Zhang, Lei Chen, Xin-wei Guo, Zhen-xing Li, Kun-feng Yan, Qi-qiang He, Hong-wei Cheng

**Affiliations:** ^1^ Department of Gastroenterology, Taixing People’s Hospital, Taixing, China; ^2^ School of Public Health, Wuhan University, Wuhan, China; ^3^ Hubei Biomass-Resource Chemistry and Environmental Biotechnology Key Laboratory, Wuhan University, Wuhan, China

**Keywords:** gastric cancer, lymph node metastasis, carcinoembryonic antigen, systemic immune-inflammation index, prognostic nutritional index

## Abstract

**Background:**

Carcinoembryonic antigen (CEA), systemic immune-inflammation index(SII), and prognostic nutritional index (PNI) are diagnostic markers for cancer, but their combined significance in gastric cancer (GC) with lymph node metastasis remains unclear. The aim of this study was to evaluate the association between these serum biomarkers and lymph node metastasis in patients with GC.

**Methods:**

Records of patients with GC were reviewed retrospectively. Univariate and multivariate logistic regression were performed to examine the association between tumor markers, serum biomarkers and lymph node metastasis in GC. Based on the results of multivariate regression, a nomogram was developed and verified.

**Results:**

Of the 395 patients aged 68.5 ± 9.1 years, 192 (48.6%) were diagnosed with lymphatic node metastasis. After adjusting for confounding factors, CEA (Odd ratio (OR):2.21; 95%CI: 1.17-3.81) and SII (OR:1.02; 95%CI: 1.01-1.04) was identified as significant risk factors, while PNI (OR:0.90; 95%CI: 0.85~0.96) was a protective factor for lymph node metastasis. The established nomogram by incorporating CEA, SII, PNI, differentiation, and tumor diameter can effectively predict lymph node metastasis in GC.

**Conclusion:**

CEA, SII, PNI, differentiation, and tumor diameter were significantly associated with lymph node metastasis in patients with GC, and the combination of CEA, SII, PNI, differentiation, and tumor diameter has a better diagnostic value than either index alone.

## 1 Introduction

Gastric cancer (GC) is a significant global public health issue, especially in East Asian countries (1). In 2020, GC was ranked the fifth most frequently diagnosed cancer, and the third leading cause of cancer-related deaths in the word, with more than 1 million cases and over 768,000 deaths (2). GC has the highest incidence and mortality rate in China. Upon diagnosis, approximately 75% of cases were already in the advanced stage of GC, with the majority accompanied by lymph node metastasis (3). Lymph node metastasis is an important influencing factor in the prognosis and the selection of optimal treatment approach among patients with GC. Therefore, it is necessary to evaluate whether lymph node metastasis occurs in patients with GC before surgery (4). At present, the clinical diagnosis of preoperative GC with lymph node metastasis mostly relies on auxiliary examinations, such as upper endoscopy, 18-fluorodeoxyglucose (FDG) positron emission tomography integrated with computed tomography (PET-CT), and abdominal ultrasonography(AUS), which cannot be reliably utilized to confirm or exclude lymph node metastasis (5, 6). Furthermore, tissue biopsy cannot be routinely used in patients with GC due to its invasiveness and high cost. Thus, it is crucial to explore inexpensive and powerful predictors for lymph node metastasis in patients with GC (7).

Previous studies have focused on tumor markers, which are quantifiable circulating substances associated with malignant diseases to predict GC (8). Among several tumor markers, carcinoembryonic antigen (CEA) and carbohydrate antigen 19-9 (CA19-9) are common tumor associated antigens (9, 10). However, previous studies regarding the association of CEA and CA19-9 with GC yield inconsistent findings (11, 12). Furthermore, only a few investigations have examined the diagnosis significance of these markers for lymph node metastasis in patients with GC.

Accumulating evidence have shown a critical role of the inflammatory microenvironment and nutritional status in the tumorigenesis and progression of GC (13). It has been found that the neutrophil-to-lymphocyte ratio (NLR) and platelet-to-lymphocyte ratio (PLR), which are indicators of systemic inflammation, can affect tumor progression by inhibiting apoptosis, promoting angiogenesis, and damaging DNA (14). Moreover, the systemic immune-inflammation index (SII), a novel inflammatory marker derived from the counts of peripheral blood neutrophils, platelets, and lymphocytes, can serve as a promising indicator of various inflammatory and immune pathways within the body and exhibit greater stability in diagnosis compared to PLR and NLR (15). Several studies showed that SII can predict the prognosis of malignant tumors, including squamous cell carcinoma (16), lung cancer, and breast cancer (17). Furthermore, it has been found that malnourished patients affect immune cell metabolism, resulting in immune dysregulation and the development of cancer. Evidence have also shown that the prognostic nutrition index (PNI), a novel index of an individual’s nutritional status, plays an important role in predicting the metastasis of various malignant tumors (18, 19). PNI can be used as a prognostic factor for survival in GC patients receiving adjuvant chemotherapy (20). Besides, PNI variability was associated with survival outcomes in gastrointestinal cancers (21). Although PLR, NLR, and CEA have been reported to be associated with GC prognosis (9, 22), their clinical correlations with GC remain controversial. Furthermore, the diagnostic values for the combined effects of these indicators in GC are unclear.

Therefore, we conducted a retrospective study to evaluate the associations of CEA, CA125, CA19-9, CA724, NLR, PLR, SII, and PNI in patients with GC, and further examined their combination on the diagnostic value of lymph node metastasis in patients with GC.

## 2 Methods

### 2.1 Ethical statement

This study was conducted in accordance with the Declaration of Helsinki and its subsequent amendments (23), and has been approved by the ethics committee of Taixing People’s Hospital (LS2023007). Due to the retrospective nature of this study without interacting with patients or using personal identifying information, the need of obtaining informed consent has been waived.

### 2.2 Study population

This study was conducted in Taixing People’s Hospital, Jiangsu Province, China. Records of 395 preoperative patients with GC who admitted to the hospital from March 2020 to March 2023 were reviewed. All patients were pathologically confirmed as GC and did not receive any therapy before admission. The inclusion criteria were as follows: (1)patients were staged according to the American Joint Committee on Cancer (AJCC)/Union for International Cancer Control (UICC) 8th edition staging system (24). (2) Only patients with complete clinical and pathological features were included in this study. Patients with preoperative radiotherapy and/or chemotherapy, antiplatelet agent therapy within the last 6 months, blood malignancies, and history of other malignant tumors were excluded.

Clinical and pathological data including gender, age tumor-node-metastasis (TNM) stage, differentiation, tumor diameter, and histological morphology were obtained from the patient’s medical records. The neutrophil, lymphocyte, platelet, and monocyte counts were collected using a routine blood test within three days before surgery. The serum albumin level was measured by hepatic function test before surgery. NLR, SII, PLR, and PNI were calculated using the following formula as follows: NLR= neutrophil counts/lymphocyte counts, SII= platelet counts × neutrophil counts/lymphocyte counts, PLR=absolute platelet count/lymphocyte count, PNI = serum albumin level (g/L) + 5 × absolute lymphocyte count (mm^3^) (25).

### 2.3 Statistical analysis

According to the laboratory reference values, the cut-off value of CEA, CA19-9, carbohydrate antigen 724 (CA724), and carbohydrate antigen 125 (CA125) was determined with the upper limit of normal defined as 5ng/ml, 27 U/ml, 6.9 U/ml, and 35 U/ml, respectively. The differences between patients with lymphatic metastasis and non-lymph node metastasis were compared by independent sample t-test for continuous variables and by chi-square test for categorical variables, respectively. Logistic regression was employed to explore the association between serum marker and lymph node metastasis in patients with GC. Significant diagnostic factors identified by univariate analysis were further assessed with multivariate logistic regression model after adjusting potential confounders using the “enter” method. Spearman correlation analysis was used to evaluate the correlations between SII, PNI, CEA and clinical variables (e.g., age, tumor diameter, differentiation, etc.) in the logistic regression model to determine whether these variables were independent. The receiver operating characteristic (ROC) curve (AUC) was used to determine the sensitivity and specificity for predicting lymph node metastasis by the parameters. The F-score was used to evaluate the performance of a classification model, measuring the accuracy of the model by balancing precision and recall (26). A nomogram was developed based on the multivariate logistic regression model. Calibration curve and decision curve analysis (DCA) were used to evaluate the performance of the nomogram model. P < 0.05 was considered statistically significant. All analyses were performed using R 4.3.

## 3 Results

**Table 1** displays the characteristics of patients. Of the 395 patients aged 68.5 ± 9.1 years, 192(48.6%) were diagnosed with lymphatic metastasis, and 282 (71.4%) were males. The average tumor diameter was 4.2 ± 2.2 cm. The results show that lymph node metastasis was significantly correlated with age (P=0.017), tumor stage, TNM, degree of differentiation and tumor diameter (P<0.001).

**Table 1:** Characteristics of patients.

Variables		Total (N=395)	Non-lymph node metastasis (N=203)	Lymph node metastasis (N=192)	*P* value
Gender	Male	282 (71.4%)	144 (70.9%)	138 (71.9%)	0.924
	Female	113 (28.6%)	59 (29.1%)	54 (28.1%)	
Age, years		68.5 ± 9.1	67.4 ± 9.1	69.6 ± 9.0	0.017
Tumor stage	T1	93 (23.5%)	86 (42.4%)	7 (3.6%)	<0.001
	T2	71 (18.0%)	56 (27.6%)	15 (7.8%)	
	T3	62 (15.7%)	21 (10.3%)	41 (21.4%)	
	T4	169 (42.8%)	40 (19.7%)	129 (67.2%)	
TNM	1	143 (36.2%)	139 (68.5%)	4 (2.1%)	<0.001
	2	102 (25.8%)	61 (30%)	41 (21.4%)	
	3	139 (35.2%)	3 (1.5%)	136 (70.8%)	
	4	11 (2.8%)	0 (0%)	11 (5.7%)	
Differentiation	Low	205 (51.9%)	78 (38.4%)	127 (66.1%)	<0.001
	Medium	148 (37.5%)	86 (42.4%)	62 (32.3%)	
	High	42 (10.6%)	39 (19.2%)	3 (1.6%)	
Tumor diameter, cm	<2.0	73 (18.5)	57 (28.1%)	16 (8.3%)	<0.001
	≥2.0	322 (81.5)	146 (71.9%)	176 (91.7%)	
Histological morphology	Adenocarcinoma	385 (97.5)	199 (98%)	186 (96.9%)	0.682
	Others	10 (2.5)	4 (2%)	6 (3.1%)	

**Figure 1:**
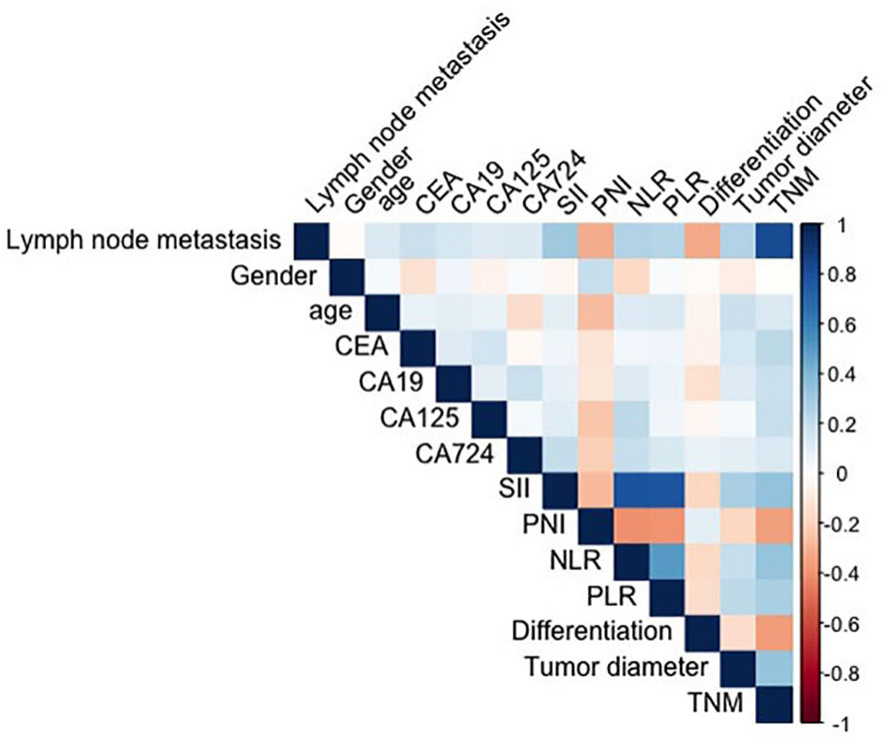
Correlation analysis heat map of SII, PNI, CEA and other clinical variables.

The spearman correlation analysis showed that the correlation coefficient between SII, PNI, CEA and other clinical variables (e.g., age, tumor diameter, differentiation, etc.) was less than 0.3, and the variables were independent. There was significant collinearity between TNM stage and lymphatic metastasis (0.817)(**Figure 1**). **Table 2** shows the univariate and multivariate logistic regression analysis of diagnostic factors in patients with lymph node metastasis. After adjusting for confounding factors, CEA (Odd ratio (OR):2.11; 95% confidence interval (CI): 1.17-3.81), SII (OR:1.02; 95%CI: 1.01-1.04), tumor diameter (OR:2.43; 95%CI: 1.23-4.79)were identified as significant risk factors that affect lymph node metastasis, while PNI (OR:0.90; 95%CI: 0.85-0.96) was associated with a lower risk for lymph node metastasis in GC.

**Table 2:** Logistic regression analysis for diagnostic factors in GC patients with lymph node metastasis.

Variables		Non-Lymph node metastasis(N=203)	Lymph node metastasis(N=192)	Univariable		Multivariable	
				OR (95%CI)	*P* value	OR (95%CI)	*P* value
Age		67.4 ± 9.1	69.6 ± 9.0	1.03 (1.00-1.05)	0.018	1.00 (0.98-1.03)	0.725
CEA(%)	Negative	177 (87.2)	139 (72.4)	2.60 (1.54-4.36)	<0.001	2.11 (1.17-3.81)	0.013
	Positive	26 (12.8)	53 (27.6)				
CA199(%)	Negative	185 (91.1)	156 (81.2)	2.37 (1.30-4.34)	0.005	1.29 (0.63-2.64)	0.484
	Positive	18 (8.9)	36 (18.8)				
CA125(%)	Negative	197 (97.0)	176 (91.7)	2.98 (1.14-7.80)	0.026	1.87 (0.50-6.97)	0.350
	Positive	6 (3.0)	16 (8.3)				
CA724(%)	Negative	126 (62.1)	96 (50.0)	1.64 (1.10-2.44)	0.016	1.62 (0.98-2.68)	0.061
	Positive	77 (37.9)	96 (50.0)				
SII		397.4 ± 277.0	677.9 ± 640.5	1.02 (1.01-1.05)	<0.001	1.02 (1.01-1.04)	0.043
PNI		48.9 ± 4.8	45.6 ± 5.0	0.87 (0.83-0.91)	<0.001	0.90 (0.85-0.96)	0.001
NLR		2.4 ± 1.4	3.9 ± 4.2	1.36 (1.18-1.57)	<0.001	0.91 (0.71-1.16)	0.518
PLR		115.6 ± 55.1	151.1 ± 73.2	1.01 (1.01-1.01)	<0.001	1.00 (0.99-1.00)	0.584
Differentiation	Low	78 (38.4%)	127 (66.1%)				
	Medium	86 (42.4%)	62 (32.3%)	0.44 (0.29-0.68)	<0.001	0.45 (0.28-0.74)	0.002
	High	39 (19.2%)	3 (1.6%)	0.05 (0.01-0.16)	<0.001	0.05 (0.01-0.18)	<0.001
Tumor diameter, cm	<2.0	57 (28.1%)	16 (8.3%)	4.29 (2.37-7.80)	<0.001	2.43 (1.23-4.79)	0.010
	≥2.0	146 (71.9%)	176 (91.7%)				

PNI, prognostic nutritional index; SII, systemic immune-inflammation index; NLR, neutrophil - lymphocyte ratio; PLR, platelet-lymphocyte ratio; CEA, Carcinoembryonic antigen; CA199, Cancer antigen 199; CA724, Cancer antigen 724; CA125, cancer antigen 125; OR, odd ratio; CI, Confidence Interval.

**Figure 2:**
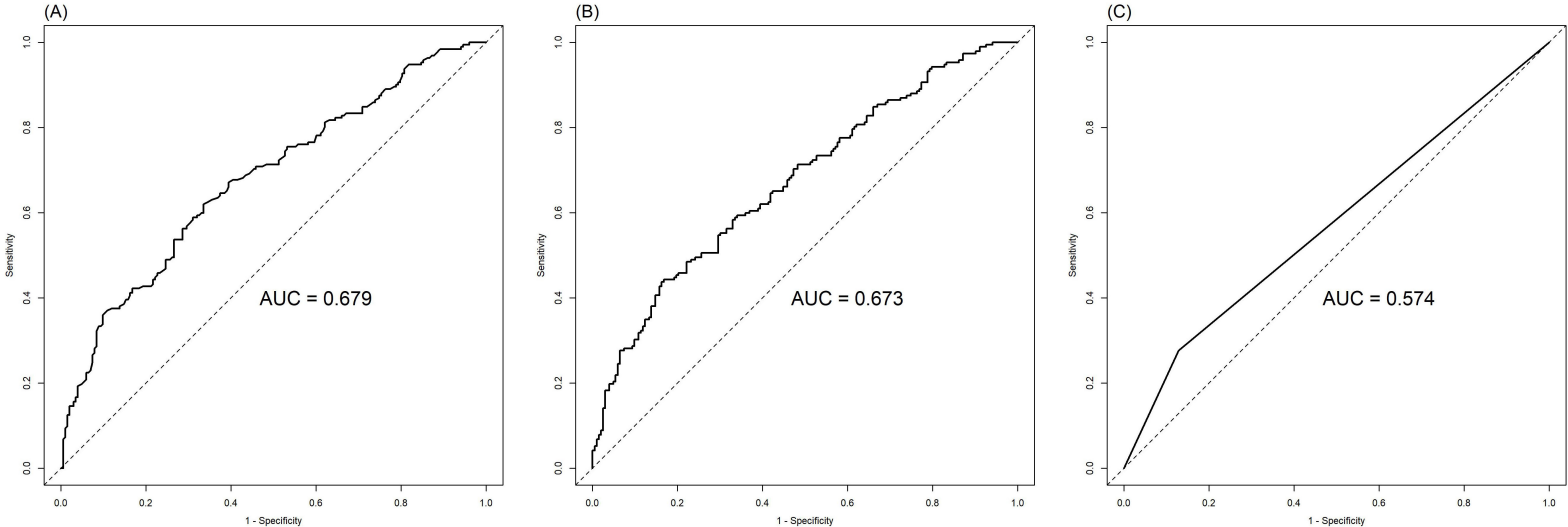
Comparison of PNI **(A)**, SII **(B)** and CEA **(C)** in the diagnosis of lymph node metastasis of GC.

**Figure 3:**
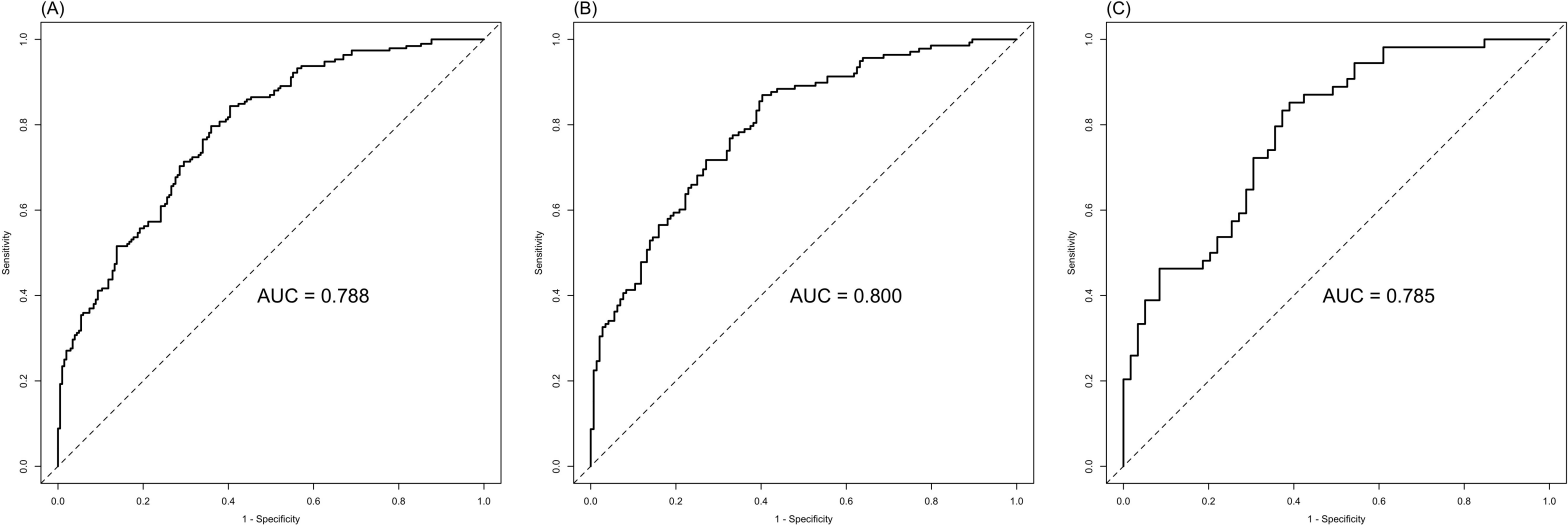
Combined SII, PNI, CEA, differentiation, and tumor diameter in the diagnosis of lymph node metastasis of GC. **(A)** ALL; **(B)** Male; **(C)** Female.

The ROC analysis of CEA, PNI, and SII is shown in **Figure 2**. The AUC of the PNI, SII, and CEA for lymph node metastasis was 0.679, 0.673, and 0.574, respectively. After applying the Bonferroni correction, the PNI, and SII demonstrated higher diagnostic accuracy compared to CEA in detecting lymph node metastasis (p<0.001). The combined diagnosis of lymph node metastasis in patients with GC using CEA, SII, PNI, differentiation, and tumor diameter yielded an AUC of 0.788, which was significantly higher than single diagnostic indicator. In male patients, the AUC for the joint diagnosis of CEA, SII, PNI, differentiation, and tumor diameter was 80.0%, while in female patients, the AUC was 78.5% (**Figure 3**). The accuracy and comprehensive performance of comprehensive model in predicting lymphatic metastasis were better than that of single index (**Table 3**). The ROC analysis of PLR, NLR, CA19-9, CA125, and CA724 is shown in **Supplementary Figure S1**. The AUC of the PLR, NLR, CA19-9, CA125, and CA724 for lymph node metastasis was 0.644, 0.649, 0.549, 0.527, and 0.560, respectively.

**Table 3:** Model performance evaluation.

Model	C-index	F-score
SII	0.673	0.543
PNI	0.679	0.617
CEA	0.574	0.391
Model1	0.788	0.705
Model2	0.800	0.693
Model3	0.785	0.703

PNI, prognostic nutritional index; SII, systemic immune-inflammation index; CEA, Carcinoembryonic antigen.

Model1:SII+PNI+CEA+differentiation+tumor diameter.

Model2: Male (SII+PNI+CEA+differentiation+tumor diameter).

Model3: Female (SII+PNI+CEA+differentiation+tumor diameter).

**Figure 4:**
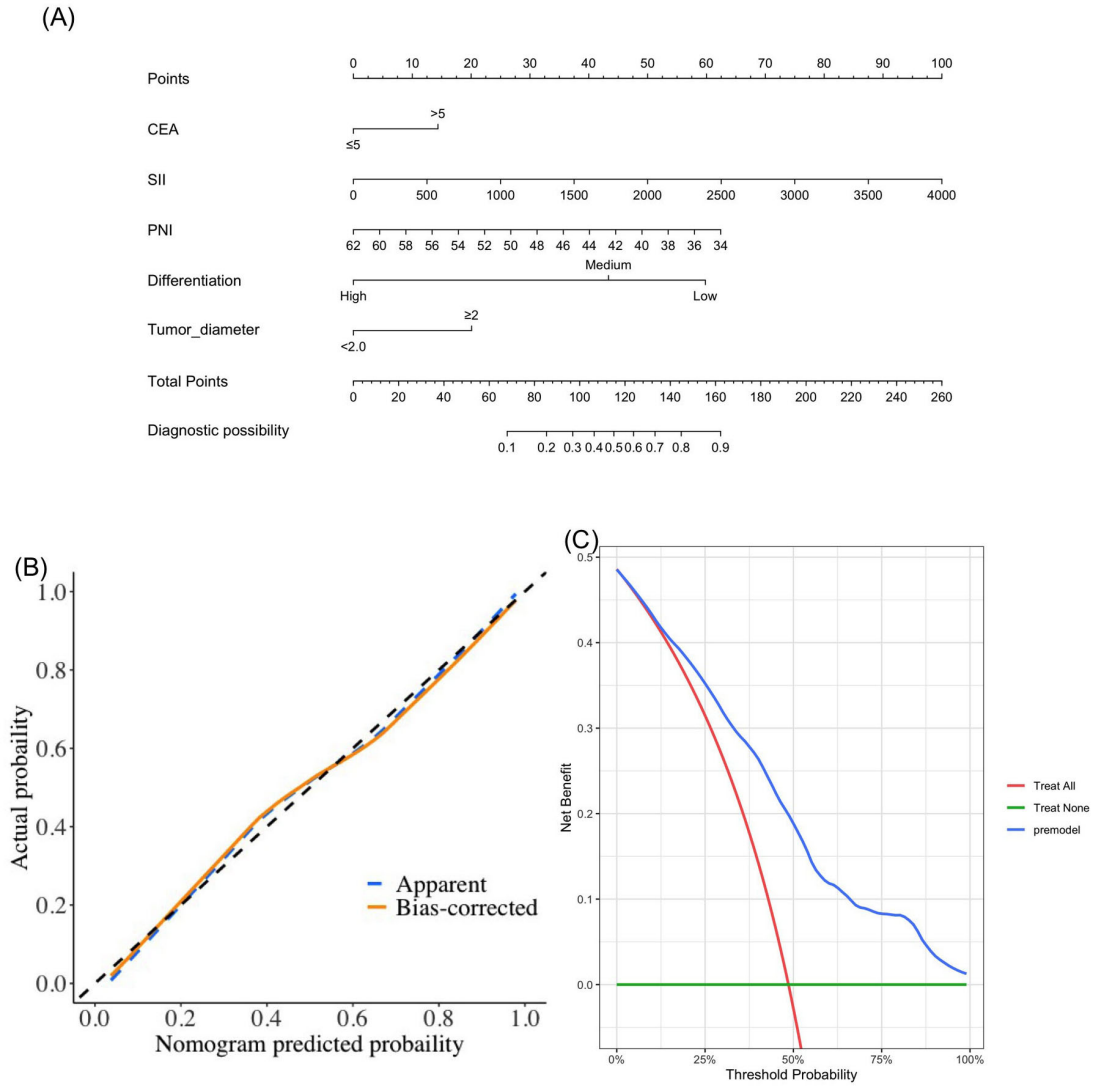
The novel nomogram **(A)**, calibration curve **(B)** and decision curve **(C)** in the diagnosis of lymph node metastasis.

Based on the results of multivariate logistic regression, CEA, SII, PNI, differentiation, and tumor diameter were included in the nomogram model (**Figure 4A**). DCA showed greater net benefits (**Figure 4B**). In addition, calibration curves also show good agreement between predictions and observations (**Figure 4C**).

## 4 Discussion

Given the asymptomatic presentation in most patients with GC prior to advanced disease stages, early GC detection assumes paramount importance (27). The main manifestation of GC metastasis is the involvement of lymph node metastasis, which is closely related to the prognosis of patients with GC. In addition, accurate assessment of preoperative lymph node status is important for the choice of GC treatment options. Therefore, it is of great significance to explore accurate preoperative diagnosis of lymph node metastasis for the formulation of treatment plans and prognosis evaluation of patients with GC (28). After a series of analyses, we used CEA, SII and PNI to develop a clinical diagnostic model for lymph node metastasis in patients with GC.

The relationship between inflammatory makers and various tumors has become the current research hotspot. Systemic inflammatory responses have been significantly associated with poor prognosis in patients with cancers (16, 29, 30). Persistent inflammatory responses can stimulate tumor growth, invasion, and metastasis (31). Of the three inflammatory factors (SII, PLR, and NLR) in the present study, SII was the only independent prognostic factor for lymph node metastasis in patients with GC in the full-adjusted statistical models. Our results were consistent with the findings by Cao et al (32). Matsubara et al. also showed that SII was an independent prognostic factor in patients with endometrial cancer and could more precisely predict survival than PLR and NLR (33). In contrast to PLR and NLR, SII consists of three peripheral blood parameters (neutrophils, platelets, and lymphocytes) (34). It can comprehensively reflect the balance of host immunity and inflammation, and dynamically obtain information about host inflammation, immune response and clinical treatment (35). Platelets deliver adenosine triphosphate into the circulation and promote tumorigenesis (36). Lymphocytes control tumor growth by secreting cytokines such as interferon-γ (IFN-γ) and tumor necrosis factor-α (TNF-α) which interact with each other (37). Neutrophil and platelets can stimulate cancer progression by producing vascular endothelial growth factor (VEGF) and interleukin-6 to enhance tumor angiogenesis and tumor cell epithelial-mesenchymal transition (38, 39).

Patients with cancers are at a high risk of malnutrition due to the proliferation of tumor cells and adverse effects of treatment (34, 40). Previous studies have found that PNI was a prognostic marker for various malignancies. In this study, PNI had a significant negative association with lymph node metastasis in patients with GC. The reason that PNI could be used as a prognostic factor for lymph node metastasis in GC lies in the fact that PNI was calculated from serum albumin concentration and peripheral blood lymphocyte count. Serum albumin may reflect the overall nutritional status of the body, and low serum albumin levels indicate poor nutrition in the host, which can negatively affect overall health, such as damage to the body’s cellular immunity, humoral immunity and phagocytic function and other defense mechanisms, increase inflammatory response, and promote tumor cell invasion function, thereby promoting the occurrence and progression of tumors (41, 42).

Previous research suggest that CEA, CA19-9, CA724, and CA125 are classical tumor markers for GC (43). In the present study, the multivariate analysis showed that CEA with a cut-off of 5ng/ml was an independent risk factor for lymph node metastasis in patients with GC, which could be used for early screening. Nevertheless, CA19-9, CA724, and CA125 were not significantly associated with lymph node metastasis in GC. CEA, a carcino-embryonic antigen, has been shown to play a role in programmed cell death and cell adhesion. Our results are similar with a previous study by Feng et al., who found that among several tumor markers including CEA, CA19-9, and CA125, elevation of CEA level was an independent prognostic factor for the poor prognosis of early GC (9). In addition, the combination of these tumor markers had a relatively low diagnostic value. Therefore, a more sensitive prediction model is warranted to be constructed in further studies.

In the present study, we found that the combination of CEA, SII, PNI, differentiation, and tumor diameter increased the AUC under the ROC to 0.788, which means that the combined index could improve the diagnosis of lymph node metastasis in patients with GC than single marker. To the best of our knowledge, this is the first study integrating tumor markers, inflammatory and nutritional indicators to diagnose lymphatic metastasis in GC. In subgroup analyses, the diagnostic sensitivity in male (AUC=0.800) was significantly higher than females (AUC=0.785). This result is consistent with the findings of a previous study showing higher diagnostic significance of systematic markers of inflammation in male than female GC patients (14). This might be explained by a more intense response immune system to lymphatic metastasis of GC cells in males than females, resulting in an increase in the proportion of neutrophils and platelets, and CEA in patients with GC is closely related to lymphatic metastasis (5). Therefore, it is warranted to include the gender factors in the early diagnosis of GC with lymph node metastasis to improve the application value of combined diagnostic indicators.

There are some limitations in this study. Firstly, blood indicators might have been affected by infection and blood circulation capacity. Secondly, we used a single-center retrospective data to build the predictive model, which may be subject to selection bias, Thus, it is necessary to conduct a large-scale prospective study with long-term follow-up from multi-centers to validate our findings. Lastly, the blood neutrophil, lymphocyte, platelet and monocyte was collected at a single time point when admission. Thus, future research should assess the dynamic changes of blood samples in multiple time points.

## 5 Conclusions

Our study demonstrated that CEA, SII, PNI, differentiation, and tumor diameter were significantly associated with lymph node metastasis in patients with GC, indicating these indices can be used for monitoring of GC patients. In addition, the established nomogram by incorporating CEA, SII, PNI, differentiation, and tumor diameter can effectively predict lymph node metastasis in GC. Therefore, the combined screening of tumor markers and blood routine indicators are recommended in clinical practice to assist in the early diagnosis of lymph node metastasis in patients with GC. Our results may provide a new biological target for the clinical diagnosis and facilitate the process of treatment of GC.

## Data Availability

Data from this study are available upon request from the corresponding author when necessary.

## Supplementary material

Supplementary Figure 1 Comparison of PLR, NLR, CA19-9, CA125, and CA724 in the diagnosis of lymph node metastasis. **(A)** PLR, **(B)** NLR, **(C)** CA19-9, **(D)** CA125, **(E)** CA724.
